# Myelin Basic Protein in Oligodendrocyte-Derived Extracellular Vesicles as a Diagnostic and Prognostic Biomarker in Multiple Sclerosis: A Pilot Study

**DOI:** 10.3390/ijms24010894

**Published:** 2023-01-03

**Authors:** Cristina Agliardi, Franca Rosa Guerini, Milena Zanzottera, Elisabetta Bolognesi, Silvia Picciolini, Domenico Caputo, Marco Rovaris, Maria Barbara Pasanisi, Mario Clerici

**Affiliations:** 1 IRCCS Fondazione Don Carlo Gnocchi, 20147 Milan, Italy; 2 Pathophysiology and Transplantation Department, University of Milan, 20122 Milan, Italy

**Keywords:** multiple sclerosis, primary progressive multiple sclerosis, extracellular vesicles, exosomes, oligodendrocytes, MOG, MBP, biomarkers

## Abstract

Approximately 15% of multiple sclerosis (MS) patients develop a progressive form of disease from onset; this condition (primary progressive-PP) MS is difficult to diagnose and treat, and is associated with a poor prognosis. Extracellular vesicles (EVs) of brain origin isolated from blood and their protein cargoes could function as a biomarker of pathological conditions. We verified whether MBP and MOG content in oligodendrocytes-derived EVs (ODEVs) could be biomarkers of MS and could help in the differential diagnosis of clinical MS phenotypes. A total of 136 individuals (7 clinically isolated syndrome (CIS), 18 PPMS, 49 relapsing remitting (RRMS)) and 70 matched healthy controls (HC) were enrolled. ODEVs were enriched from serum by immune-capture with anti-MOG antibody; MBP and MOG protein cargoes were measured by ELISA. MBP concentration in ODEVs was significantly increased in CIS (*p* < 0.001), RRMS (*p* < 0.001) and PPMS (*p* < 0.001) compared to HC and was correlated with disease severity measured by EDSS and MSSS. Notably, MBP concentration in ODEVs was also significantly augmented in PPMS compared to RRMS (*p* = 0.004) and CIS (*p* = 0.03). Logistic regression and ROC analyses confirmed these results. A minimally invasive blood test measuring the concentration of MBP in ODEVs is a promising tool that could facilitate MS diagnosis.

## 1. Introduction

Multiple sclerosis (MS) is an immune-mediated demyelinating disease of the central nervous system (CNS) [1] that predominantly affects young adults. The disease has a prevalence of 50–300/100.000 people, and about 2–3 million people are estimated to live with MS globally [2]. Proteins within the CNS are the targets of autoimmune responses in MS. Thus, myelin is a multilamellar sheath necessary to insulate neurons’ axons and increase the rate at which action potentials are passed along; it is formed by the elaboration of oligodendrocyte processes around axons [3]. The main proteinaceous components of myelin, myelin basic protein (MBP), myelin oligodendrocyte glycoprotein (MOG), and proteolipid protein (PLP), are effective autoantigens and targets of autoimmunity in MS [4,5,6].

MS is characterized by a highly variable clinical course that spans from minimally disabling to severe forms resulting in progressive, irreversible clinical and cognitive deficits and limited response to therapy [7]. Relapsing remitting (RR) MS patients are patients who experience episodes of neurological dysfunction with or without residual disability. About 15–30% of RRMS patients will develop progressive disability and become secondary progressive (SP) MS patients. Finally, about 15% of patients develop primary progressive (PP) MS, a worsening neurologic function from the onset of symptoms, without relapses or remissions [8]. The identification of easily reproducible and minimally invasive blood-based biomarkers would facilitate the early classification of MS patients into these different clinical subtypes, allowing the optimization of therapeutic and rehabilitative strategies.

Extracellular vesicles of brain origin can cross the blood brain barrier (BBB) and can be isolated from peripheral blood [9]; their cargoes protein could function as biomarkers of pathological conditions. Myelin oligodendrocyte glycoprotein (MOG) is expressed at the RNA level predominantly in oligodendrocytes (https://www.proteinatlas.org/ENSG00000204655-MOG/single+cell+type (accessed on 16 September 2022)) and can be used to identify oligodendrocyte-derived extracellular vesicles (ODEVs) [10]. Using a newly developed protocol, we enriched ODEVs from the serum of subjects with clinically isolated syndrome (CIS), i.e., a single episode of CNS dysfunction suggestive of MS, RRMS, PPMS, and healthy controls (HC) to verify whether ODEVs MBP and MOG concentrations could be used as a diagnostic and prognostic biomarker for MS.

## 2. Results

### 2.1. Cohort Characteristics

The clinical and demographic characterization of all the individuals enrolled in the study is presented Table 1. A total of 67 MS patients (49 RRMS and 18 PPMS) had a confirmed diagnosis of MS according to the 2017 McDonald diagnostic criteria [11]. Seven subjects were defined as CIS. There were some statistical differences in gender distribution among groups (see Table 1). When MS patients were considered as a unique group (RRMS + PPMS), the differences lapsed. Age at onset was significantly higher in PPMS compared to RRMS (*p* < 0.01). As expected, both expanded disability status scale (EDSS) and Multiple Sclerosis Severity Score (MSSS) scores were higher in PPMS compared to RRMS and CIS patients (see Table 1). Finally, the mean age of all the groups of individuals was comparable.

### 2.2. HLA-DRB1*15 Genotyping

The presence of the HLA-DRB1*15 allele either in heterozygosity or homozigosity was evaluated in all individuals. HLA-DRB1*15 was significantly more often seen in MS patients (CIS + RRMS + PPMS) compared to HC (*p* = 2.8 × 10^−5^, Odds ratio (OR) = 10.58, 2.82–67.99 95% confidence interval (CI)), confirming the association between the disease and this allele.

### 2.3. ODEVs Characterization

**Figure 1:**
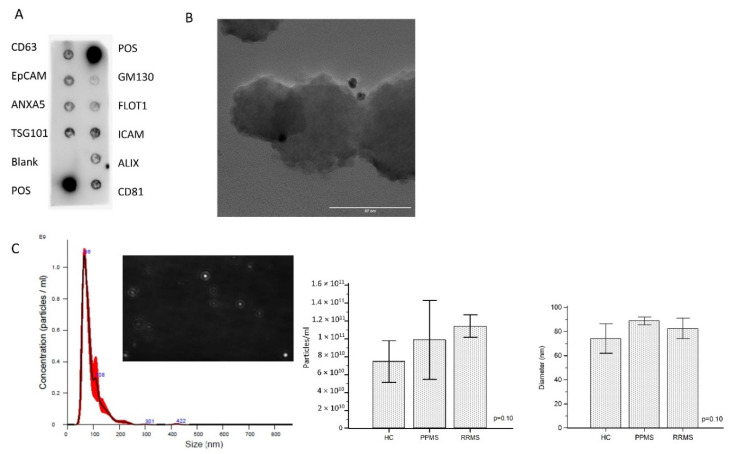
**ODEVs characterization.** (**A**): Exo-Check™ Exosome Antibody Array on an exemplificative ODEVs lysate. In the image are visible exosomal associated markers: FLOT1 (flotillin-1), ICAM1 (intercellular adhesion molecule 1), ALIX (programmed cell death 6 interacting protein), CD81 and CD63 (tetraspanins), EpCAM (epithelial cell adhesion molecule), ANXA5 (annexin A5), TSG101 (tumor susceptibility gene 101) and controls (2 positive assay control, negative control: blank and GM130: cis-golgi matrix protein: control for cellular contamination). (**B**): Immuno-gold (OMGp antigen detected) TEM micrograph of an exemplificative ODEVs preparation. Scale bar: 100 nm. (**C**): Representative size distribution graph of nanoparticle tracking analysis (NTA) that shows size and concentration of enriched ODEVs in a sample from an RRMS patient; a frame of the video is also shown. Mean ODEVs concentration (particles/mL) ± SD and mean ODEVs diameter (nm) ± SD obtained by NTA analysis from five ODEVs samples from the three conditions (HC, PPMS, and RRMS). ANOVA tests *p* values are reported.

Enriched ODEVs were characterized according to the international society of Extracellular vesicles (ISEV) directions regarding minimal information for studies of extracellular vesicles (MISEV) [12]. A representative developed membrane of Exo-Check Exosome Antibody Array is shown in Figure 1A. Exosomal-associated markers (CD63, CD81, ALIX, FLOT1, ICAM1, EpCam, ANXA5, and TSG101) were all present in enriched ODEVs lysate (ODEVs), as were the two positive controls. The absence of the GM130 cis-Golgi marker evidences absence of cellular contamination. The dimensions and morphology of isolated extravesicles was analyzed by immuno-gold transmission electron microscopy (TEM). A heterogeneous population of extravesicles, in terms of size and shape, was detected. Immunogold labeling with anti OMG mAb confirmed ODEVs enrichment: black punctate regions indicate a positive staining on ODEVs membrane surface (Figure 1B). Quantitative measurements of enriched ODEVs were conducted in five representative samples from the three groups: HC, PPMS, and RRMS. The mean concentration of NDEVs after an ANOVA test resulted in comparable among groups (HC = 6.3 × 10^10^ particles/mL, standard deviation (SD): 2.6 × 10^10^ particles/mL; PPMS = 6.0 × 10^10^ particles/mL, SD: 2.2 × 10^9^ particles/mL; RRMS = 1.1 × 10^11^ particles/mL, SD: 1.2 × 10^10^ particles/mL) (*p* = 0.10) (Figure 1C). NDEVs’ mean dimension was comparable among groups as well (HC = 74.2 ± 12.3 nm; PPMS = 88.9 ± 3.2 nm; RRMS = 82.5 ± 8.5 nm) (*p* = 0.10) (Figure 1C). 

### 2.4. MBP Concentration in Enriched ODEVs Is Increased in MS Patients

**Figure 2:**
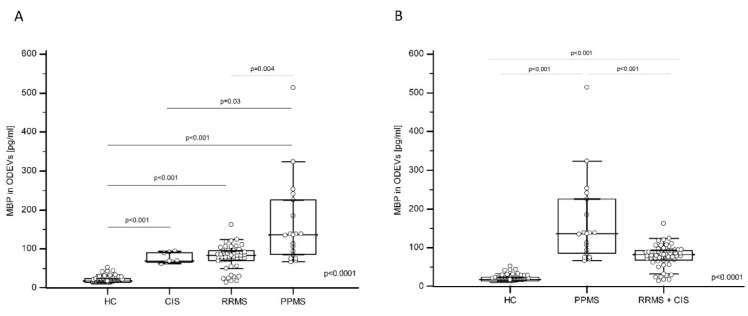
**MBP in enriched ODEVs in HC, CIS, RR-MS, and PP-MS.** (**A**): Multiple comparison graphs of MBP concentration in enriched ODEVs, respectively in HC, CIS, RRMS, and PPMS subjects; all data are plotted, and median and interquartile range (IQR) are reported. The reported global *p* values of the differences between the groups of subjects was calculated by Kruskal–Wallis test for non-parametric distributions. *p* values of post hoc Dwass–Steel–Critchlow–Fligner for pairwise comparisons are also reported. (**B**): Multiple comparison graphs of MBP concentration in enriched ODEVs, respectively in HC, (CIS + RRMS) and PP-MS subjects; all data are plotted; median and interquartile range (IQR) are reported. The reported global *p* values of the differences between the groups of subjects was calculated by Kruskal–Wallis test for non-parametric distributions. *p* values of post hoc Dwass–Steel–Critchlow–Fligner for pairwise comparisons are also reported.

The MBP and MOG concentration in enriched ODEVs from HC, CIS, RRMS, and PPMS was analyzed by a sandwich enzyme-linked immunosorbent assay (ELISA) and was not normally distributed as per the results of Kolmogorov–Smirnov analyses. The Kruskal-Wallis test was then applied; results showed the presence of a statistically significant difference in MBP concentration among groups (*p* < 0.0001) (Figure 2A). In particular, the Dwass–Steel–Critchlow–Fligner test for pairwise comparisons indicated that the MBP concentration was significantly lower in HC (median: 17.85 pg/mL, interquartile range (IQR): 14.00–24.39 pg/mL) compared to CIS (median: 78.73 pg/mL, IQR: 65.78–91.20 pg/mL; *p* < 0.001 vs. HC), RRMS (median: 83.76 pg/mL, IQR: 71.05–95.03 pg/mL; *p* < 0.001 vs. HC) and PPMS (median: 136.44 pg/mL, IQR: 86.32–225.67 pg/mL; *p* < 0.001 vs. HC) (Figure 2A). Moreover, the MBP concentration in ODEVs was significantly increased in PPMS compared to RRMS (*p* = 0.004) and CIS (*p* = 0.03) (Figure 2A), whereas no significant differences emerged when CIS was compared to RRMS. Interestingly, if CIS subjects and RRMS patients were grouped (RRMS + CIS), the difference in ODEVs MPB concentration between PPMS (median: 136.44 pg/mL, IQR: 86.32–225.67 pg/mL) and RRMS + CIS (median: 82.16; IQR: 68.09–93.51 pg/mL) patients was even more evident (*p* < 0.01) (Figure 2B).

A logistic regression analysis was performed next, considering the disease phenotype (CIS, RRMS, and PPMS) or the condition of HC as the dependent variable, and MBP concentrations in ODEVs, as well as gender, age, and presence of at least one HLA-DRB1*15 allele as covariates. The model was applied on the overall group of the 136 subjects enrolled in the study. Results confirmed the significant contribution of ODEVs MBP concentration in discriminating between MS and healthy condition (overall model fit: χ^2^ = 113.61; *p* < 0.0001); the other covariates were not statistically significant. The model had the power to correctly classify 90.83% of cases. The same logistic regression was applied considering the clinical diagnosis of either RRMS + CIS or PPMS as a dependent variable and the same above-mentioned variables as covariates. The contribution of MBP concentration in ODEVs was statistically significant (*p* = 0.003), as were gender (*p* = 0.05) and age (*p* = 0.004), in discriminating between the two diagnoses (overall model fit: χ^2^ = 40.91; *p* < 0.0001).

In contrast with these results, the MOG protein was detected in enriched ODEVs from both MS patients and HC, but no statistically significant differences among groups could be observed (data not shown).

### 2.5. ROC Curve Analysis

**Figure 3:**
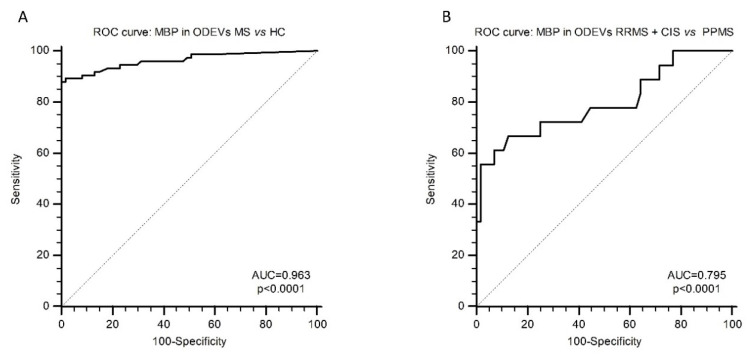
**ROC curve analysis.** (**A**): ROC curves of MBP in enriched ODEVs: HC vs. MS (CIS + RRMS + PPMS). AUC and *p* value are reported. (**B**): ROC curves of MBP in enriched ODEVs: PPMS vs. (CIS + RRMS). AUC and *p* value are reported.

Receiver operating characteristic (ROC) curve analysis was applied next to evaluate the possible diagnostic value of ODEVs MBP concentration. When MBP in ODEVs was analyzed in HC vs. MS patients (considering together CIS, RRMS, and PPMS) an ROC area under the curve (AUC) = 0.963 (0.916–0.988 95% confidence interval (CI)) with 97.84% sensitivity and 100.00% specificity (*p* < 0.0001) was obtained (Figure 3A). When ROC curve analysis was applied considering the clinical diagnosis (RRMS + CIS vs. PPMS), an ROC AUC = 0.795 (0.685–0.880 95% CI) with 66.67% sensitivity and 87.50% specificity (*p* < 0.0001) was obtained (Figure 3B).

### 2.6. Correlation with Clinical Scores

**Figure 4:**
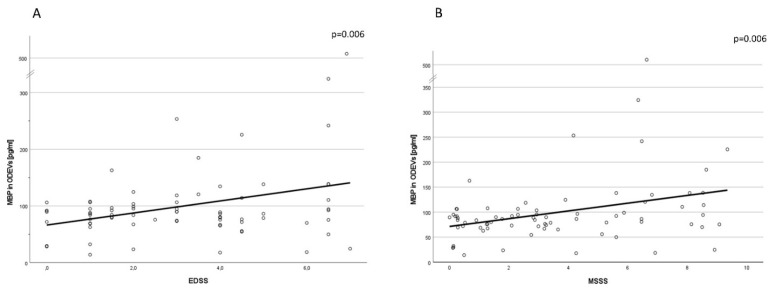
(**A**): **Correlation between MBP in ODEVs and clinical scales**. Bivariate Pearson’s correlation between MBP concentration in enriched ODEVs and EDSS. (**B**): Bivariate Pearson’s correlation between MBP concentration in enriched ODEVs and MSSS.

Pearson’s correlations showed that MBP concentration in ODEVs was correlated both with EDSS (*p* < 0.01, coefficient of correlation = 0.32, 0.09–0.51 95% CI) and MSSS (*p* < 0.01, coefficient of correlation = 0.32, 0.09–0.51 95% CI) scores (Figure 4A,B).

## 3. Discussion

In the present case-control pilot study, we show that quantification of MBP as a cargo protein inside enriched oligodendrocyte extracellular vesicles (ODEVs) isolated from serum allows identification of MS, as it discriminates between MS patients and healthy controls in a highly sensible and specific way. Moreover, and more interestingly, the amount of MBP in ODEVs can differentiate between MS patients with a clinical diagnosis of either PPMS or RRMS. Finally, we show that MBP concentration in ODEVs is also positively correlated with MS severity, as evaluated by the EDSS and MSSS scales.

The spectrum of MS disease spans from non-disabling forms to forms characterized by progressive, irreversible, clinical, and cognitive deficits and limited response to standard treatment [7]. PPMS has unfortunately a poor prognosis, and therapies approved for RRMS have little or no benefits in PPMS patients, even if treatment efficacy for two molecules—rituximab [13] and ocrelizumab [14]—has been reported. A prompt diagnosis of the different clinical MS phenotypes in the initial stages of disease could be of utmost importance for therapeutic decisions. Knowing more in advance the progressive prognosis of patients could aid in treatment and rehabilitation decision-making and could help to minimize costs for the sanitary system [15]. The diagnosis of MS is currently based on the integration of clinical, imaging, and laboratory findings [11]; age, sex, spinal cord lesions, and the extent of brain abnormalities on MRI are predictors of outcome across the different MS clinical phenotypes. Notably, though, easily accessible and non-invasive biomarkers to diagnose the disease or to characterize it are not currently available; ODEVs MBP quantification could be a useful tool.

MBP was firstly isolated in the early 1960s and constitutes 30% of total CNS myelin proteins, [16] it is the most widely studied myelin protein in MS as it is the main target of MS-associated autoimmune responses. The added value of testing MBP in CSF for MS diagnosis was suggested to be low [17], but recently it was reported as a potential biomarker of disability progression in SPMS [18]. Oligodendrocytes are known to secrete large quantities of exosomes expressing myelin proteins in vitro [19], and exosomes of CNS origin are known to cross the BBB [9]. Galazka et al. recently reported that MOG and other myelin proteins can be detected in serum exosomes of both MS patients, especially during clinical exacerbation, and non-MS subjects [20]. They analyzed total EVs preparations obtained from serum instead of enriched ODEVs and compared the concentrations of myelin proteins, including MBP and MOG, in RRMS, secondary progressive (SP)MS and HC. Results showed that MOG was increased in EVs from secondary progressive (SP)MS and RRMS during clinical exacerbations compared to healthy controls; notably, no differences in MBP concentration were detected among groups. The apparent discrepancy between Galazka’s and our results could be due to the fact that in our case, the source of myelin proteins was enriched ODEVs instead of total serum EVs. Moreover, the enrolled groups of MS patients were different in the two studies: We did not study SPMS and RRMS patients during clinical relapse; Galazka, on the other hand, did not analyze PPMS patients. Galazka et al. also found that serum exosomes were able to induce the proliferation of MOG-recognizing transgenic TCR T lymphocytes. This observation led to the hypothesis that the augmented amounts of CNS myelin proteins and peptides seen in EVs of MS patients could result in the maintaining and the amplification of autoimmune reactions and/or the modulation of immune effector mechanisms in response to the spread of such autoantigens [20]. Data herein confirm that CNS proteins can be detected in ODEVs isolated from serum and indicate that the measurement of one of such proteins, MBP, could have a diagnostic and prognostic value.

Interestingly, and although needing to be confirmed in larger cohorts, results showed that the MBP concentration in ODEVs is comparable in CIS and RRMS patients. Because CIS can, but does not always evolve into RRMS over time, it will be interesting to verify in longitudinal studies whether the MBP concentration in ODEVs will be able to predict which CIS patients will develop RRMS.

The study has some limits: first of all, the small sample size. Moreover, we do not know if different pharmacological treatments could affect MBP concentrations in ODEVs.

It will be necessary to confirm these results in larger cohorts of patients; it will be interesting to design follow-up studies on patients in the initial phases of the disease in order to verify whether MBP concentration in ODEVs will allow the precocious identification of different clinical MS phenotypes.

## 4. Material and Methods

### 4.1. Study Cohort

A total of 136 individuals were included in this case-control study. Sixty-seven MS patients and 7 individuals with a CIS diagnosis were enrolled by the Multiple Sclerosis Unit of the IRCCS Fondazione Don Carlo Gnocchi, Milan. MS patients were classified by a neurologist according to the revised McDonald’s diagnostic criteria [11] in two clinical subgroups: 18 of them were diagnosed as being affected by PPMS; the remaining 49 patients had a diagnosis of RRMS and they were all in remission phase at the time of blood collection. At the time of enrollment, demographic and clinical features (age, age at onset, disease duration, EDSS [21], pharmacological treatments) were registered in a pseudo anonymized database. MSSS was calculated as described by Roxburgh [22]. A group of 70 healthy subjects were enrolled as healthy controls (HC) among hospital staff. Exclusion criteria for HC were any autoimmune and/or neurological conditions and age <35 years in order to reduce the risk to introduce false-negative subjects. Informed consent was obtained from all the individuals prior to inclusion in the study. The study was conducted according to the guidelines of the Declaration of Helsinki and was approved by the institutional review board of the Don Carlo Gnocchi ONLUS Foundation, Milan (Protocol number: #11_27/06/2019). Demographic and clinical data of study population are reported in Table 1.

### 4.2. Serum Collection

Blood samples collection and preparation were conducted in a standardized way: 25 mL of peripheral whole blood was collected using a serum separator tube (SST II Advance, BD Vacutainer^®^). Samples were allowed to clot for 10–20 min at room temperature. Tubes were then centrifuged at 1500× *g* for 10 min, and serum was collected, aliquoted, and stored at −80 °C.

### 4.3. ODEVs Enrichment

ODEVs were enriched from 250 µL of serum by adjusting a previously published double step method [23]. Briefly, after addition of 150 µL of calcium- and magnesium-free Dulbecco balanced salt solution (PBS) and 15 µL of Halt™ Protease and Phosphatase Inhibitor Cocktail (Thermo Fisher scientific, Waltham, MA, USA) samples were centrifuged at 3000× *g* for 30 min at 4 °C; 126 µL of ExoQuick^®^ exosome precipitation solution (System Bioscience, Palo Alto, CA, USA) was added to supernatants and the mix was centrifuged at 1500× *g* for 30 min at 4 °C. Pellets were suspended in 350 µL of PBS, 50 µL of 3% bovine serum albumin (BSA), and 2 µg of anti-oligodendrocyte myelin glycoprotein (MOG) biotinylated antibody (Bioss antibodies, Boston, MA, USA). After an incubation of 1 h at room temperature, 10 µL of Pierce™ Streptavidin UltraLink™ Resin and 40 µL of 3% BSA were added; samples were then incubated for 30 min at room temperature. After centrifugation at 800× *g* for 10 min at 4 °C and removal of supernatants, pellets were suspended in 100 µL of 0.05 M glycine-HCl (pH 3.0) and vortexed 10 s. After centrifugation at 4000× *g* for10 min at 4 °C, 25 µL of 10% BSA, 10 µL of Tris-HCl and 15 µL of Halt™ Protease and Phosphatase Inhibitor Cocktail (Thermo Fisher scientific, Waltham, MA, USA) was added. Twenty µL of intact enriched ODEVs preparations was frozen and stored at 80 °C for downstream applications, the remaining extravesicles were lysed by adding 365 µL of mammalian protein extraction reagent (M-PER™, Thermo Fisher scientific, Waltham, MA, USA), and 15 µL of protease/phosphatase inhibitors. Lysates of enriched ODEVs were subjected to 2 freeze-thaw cycles and stored at −80 °C.

### 4.4. ODEVs Characterization

Lysates of enriched ODEVs were tested for the expression of canonical exosome markers by a commercial kit based on Western blotting (Exo-check Exosome Antibody Array, System Bioscience, Palo Alto, CA, USA) following manufacturer’s instructions. It consists of 12 pre-printed spots, including 8 antibodies for known exosome markers (CD63, CD81, ALIX, FLOT1, ICAM1, EpCam, ANXA5 and TSG101), and 4 controls (two positive control signals, a background control, and the GM130 cis-Golgi marker that monitors for cellular contamination). Membranes were developed using the Clarity Max Western ECL Substrate (Bio-Rad, Hercules, CA, USA) and imaged by ChemiDoc™ Gel Imaging System (Bio-Rad, Hercules, CA, USA).

Immuno-gold TEM imaging was performed as follows: 5 µL of exosomes suspensions was adsorbed on 200 mesh thin-film formvar-carbon coated TEM grids for 10 min and excess was blotted on filter paper. Two washing steps on 50-μL drops of wash buffer (0.1% BSA in PBS) were performed. TEM grids were then incubated in a wet chamber at room temperature for 3 h on 50 μL drops containing Primary Antibody (anti OMG for ODEVs) diluted 1:100 in wash buffer. Additional washing steps on 50-μL drops of wash buffer were performed. TEM grids were then incubated for 1 h on 30 μL drops of 1:50 dilutions of anti-rabbit or anti-mouse IgG-gold conjugate (10-nm particle size) in wash buffer. Washing steps on five drops of wash buffer followed by five washes on water drops were performed. Negative staining was done on 1% filtered aqueous solution of Uranyl Acetate drops for 10 s before drying. TEM micrographs were acquired by means of the JEOL JEM 2100Plus Transmission Electron Microscope (JEOL, Tokyo, Japan) operating with an acceleration voltage of 200 kV and equipped with an 8 megapixel Gatan (Gatan, Pleasanton, CA, USA) Rio Complementary Metal-Oxide-Superconductor (CMOS) camera.

To evaluate size and concentration of ODEVs nanoparticles, tracking analysis (NTA) was performed for five representative samples from each group (HC, PPMS, and RRMS) on a NanoSight NS300 device equipped with a blue 488 nm laser, a flow-cell top-plate, and a syringe pump to enable analysis in constant flow (Malvern Panalytical, Malvern, UK). Samples were diluted 1:250 in 1X filtered PBS. Three videos of 60 s were acquired per sample. Mean sizes were calculated by integrating the data from three records. Data were analyzed using the NTA software *v.* 3.4 with the detection threshold 5 to track as many particles as possible with minimal background.

### 4.5. Immunoenzymatic Dosages

MBP and MOG protein concentration in undiluted lysates from enriched ODEVs was measured by sandwich ELISA using commercial kits (cat n°: MBS2502574 and MBS928110 respectively, MyBiosource, San Diego, CA, USA), according to manufacturers’ instructions. Standard curves and samples were run in duplicate.

### 4.6. HLA-DRB1*15 Genotyping

The presence of HLA-DRB1*15 alleles, either in heterozygous or homozygous form, was inferred by the genotyping of the tag SNP rs3135388 [24] by allelic discrimination real-time PCR with the TaqMan™ C__27464665_30 probe (Thermo Fisher scientific, Waltham, MA, USA). PCR consisted of a hot start at 95 °C for 10 min followed by 40 cycles of 94 °C for 15 s and 60 °C for 1 min. Fluorescence detection took place at 60 °C. Assays were performed in 10 μL reactions using 1 μL of DNA at 50 ng/μL, using TaqMan™ Genotyping Master Mix (Thermo Fisher scientific, Waltham, MA, USA) on 96-well plates using a CFX96 instrument (Bio-Rad, Hercules, CA, USA). Control samples representing all possible genotypes and a negative control were included in each reaction. 

### 4.7. Statistical Analysis

Differences in demographic and clinical variables between HC, CIS, RRMS, and PPMS patients were evaluated by Chi-square and Student’s *t* test. Kolmogorov–Smirnov test showed that both MBP and MOG protein concentrations were not normally distributed in the present cohort. Non-parametric Mann–Whitney U test or Kruskal–Wallys test were then performed to compare MBP and MOG proteins’ concentration between different groups. Post hoc Dwass–Steel–Critchlow–Fligner test was applied for pairwise comparisons. NTA results (ODEVs concentration and dimension) were analyzed by ANOVA tests in order to highlight possible differences amongst groups of patients. The discriminatory ability of enriched ODEVs biomarkers to distinguish between different groups was analyzed by using receiver ROC analyses with calculation of the AUC, sensitivity and specificity as well as 95% CI. In general, an AUC between 0.7 and 0.8 is considered “acceptable”, an AUC between 0.8 and 0.9 is considered “excellent”, and more than 0.9 is considered “outstanding” [25]. Logistic regressions were applied considering the different conditions of illness as dependent variable, and ODEVs MBP concentrations, age, gender, and HLADRB1*15 positivity as covariates. Bivariate Pearson’s correlations were applied in order to investigate possible correlations between biological biomarkers and clinical features of patients. In all cases, differences were considered statistically significant when *p* ≤ 0.05. MedCalc^®^ software (MedCalc^®^, 14.10.2, Belgium), SPSS software (v.27, IBM, USA), jamovi (version 2.2, https://www.jamovi.org (accessed on 6 October 2022)) and OpenEpi (https://www.openepi.com (accessed on 6 October 2022)) were used for statistical analyses.

## Figures and Tables

**Table 1:** Study cohort demographic and clinical characteristics.

Study Cohort				
	CIS	RRMS	PPMS	HC
Number (*n*)	7	49	18	62
Females (*n*, %)	2 (28.6) ^&^	32 (65.3) ^&,¥^	5 (38.5) ^¥,#^	32 (51.6) ^#^
Age (years ± SD)	44.14 ± 7.10	45.80 ± 9.46	54.00 ± 12.53	51.14 ± 12.29
Disease duration (years ± SD)	10.14 ± 6.69	14.71 ± 9.02	12.44 ± 11.98	n.a.
Age at onset (years ± SD)	34.00 ± 3.37	31.08 ± 9.37 *	41.56 ± 12.26 *	n.a.
EDSS (mean ± SD)	1.57 ± 1.51 ^$^	2.62 ± 1.94 ^£^	5.08 ± 1.40 ^$,£^	n.a.
MSSS (mean ± SD)	1.38 ± 1.25 °	2.72 ± 2.32 ^§^	6.97 ± 2.06 °^,§^	n.a.

^&^*p* = 0.01; ^¥^ *p* < 0.01; ^#^ *p* = 0.04; * *p* < 0.01; ^$^ *p* < 0.001; ^£^ *p* < 0.001; ° *p* < 0.001; ^§^ *p* < 0.001.

## Data Availability

The data presented in this study are available on request from the corresponding author.
